# The 90th Birthday of Professor Raphael Mechoulam, a Top Cannabinoid Scientist and Pioneer

**DOI:** 10.3390/ijms21207653

**Published:** 2020-10-16

**Authors:** Roger G. Pertwee

**Affiliations:** School of Medical Sciences, The Institute of Medical Sciences, University of Aberdeen, Aberdeen AB25 2ZD, UK; rgp@abdn.ac.uk; Tel.: +1-224-437-404


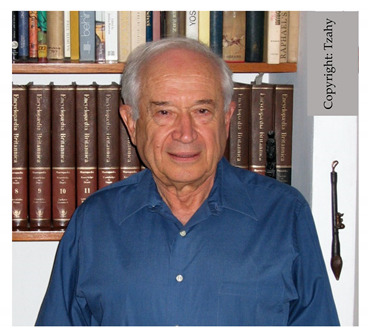


On the 5th November 2020, Professor Raphael Mechoulam, who is widely recognized as one of the greatest scientists in the field of cannabinoid research, and who is still an active researcher, will be celebrating his 90th birthday. Raphael Mechoulam was born in Bulgaria but, together with his Jewish parents, emigrated to Israel in 1949. He obtained an MSc in biochemistry in 1952 from the Hebrew University of Jerusalem, and subsequently a PhD in chemistry from the Weizmann Institute in Rehovot, near Tel Aviv. He then carried out postdoctoral research at the Rockefeller Institute in New York, before returning to The Hebrew University of Jerusalem in 1965, where he was appointed as Associate Professor in 1972 and as Professor of Medicinal Chemistry in 1975. It is at The Hebrew University where he began his prestigious cannabinoid research career, and where he is still located. Just a few of his many great cannabinoid-related achievements are highlighted below.

When Raphael Mechoulam began his cannabinoid experiments in the 1960s, a considerable amount of research, starting from the mid-19th century, had already been done on the chemistry of the cannabis plant, including the elucidation of the structure of cannabinol, which is just one of the many cannabinoid (phytocannabinoid) constituents of cannabis [1]. However, it was Mechoulam and some of his Hebrew University collaborators who, in a series of papers starting in 1963 [2,3,4,5], first reported the isolation, structure elucidation, stereochemistry and activity of Δ^9^-tetrahydrocannabinol (Δ^9^-THC), which is the main psychoactive constituent of cannabis, and was originally named Δ^1^-THC [2,3]. They also reported the isolation and structural elucidation and chemical synthesis of numerous additional cannabinoids, such as cannabidiol [4], cannabigerol, cannabichromene and some cannabinoid carboxylic acids; these are achievements that, for example, greatly facilitated the determination of the pharmacological actions of cannabis and its phytocannabinoids [1]. Importantly, Mechoulam edited and also contributed chapters, as did I, to a book entitled “Marijuana” [6] which is an excellent and very useful historical source of information about the then state-of-the-art information regarding cannabis/cannabinoid chemistry, pharmacology, metabolism and clinical effects up to 1973, and also about information that paved the way to important major new discoveries about cannabinoids that were made by Mechoulam and others after 1973 (e.g., see below).

For nearly two decades after the identification of THC, its mechanisms of action were believed to be entirely “non-specific”. However, in the 1980s, findings obtained by several research groups suggested that this might not be true. These included findings obtained by Mechoulam and his collaborators showing that certain cannabinoids display stereoselectivity [7]. Such findings encouraged a search for a cannabinoid receptor in mammalian tissues, and this search led to the discovery of two G protein-coupled cannabinoid receptors [7,8]; the first (CB_1_) was discovered between 1988 and 1990 [9,10], and the second (CB_2_) was discovered in 1993 [11]. The evidence obtained in the late 1980s that mammalian tissues express the CB_1_ receptor immediately prompted searches for a chemical produced by these tissues that can activate this receptor. The race to discover such an “endocannabinoid” was won by Mechoulam. He led research that provided convincing evidence that (i) *N*-arachidonoyl ethanolamine, which he and his collaborators named anandamide, is an endogenously produced compound that can activate the CB_1_ receptor [12], and (ii) that 2-arachidonoylglycerol is also a cannabinoid receptor-activating endocannabinoid [13]. Very wisely, given the lipophilicity of phytocannabinoids such as THC, Mechoulam decided to look for endocannabinoids among endogenous lipophilic compounds rather than endogenous peptides, even though certain peptides, named endorphins, had already been found, here in Aberdeen, to serve as endogenous activators of opioid receptors [14]. Other lipophilic endocannabinoids were also subsequently discovered [15].

The discovery of the endocannabinoid system greatly boosted cannabinoid research especially when evidence subsequently emerged that this system plays important protective roles in several serious disorders both within and outside the central nervous system [16], raising the possibility that some disorders could be treated with drugs that enhance the levels of protectively released endocannabinoids within and/or without the central nervous system by (i) raising their levels via inhibition of their metabolism or cellular reuptake and/or (ii) strengthening their ability to activate cannabinoid receptors by administering a positive allosteric modulator of these receptors [16,17,18,19].

Among Raphael Mechoulam’s many other achievements is the design and synthesis of numerous important novel cannabinoids that serve as valuable experimental tools or have important therapeutic potential. These synthetic compounds include, amongst many others, HU-210, HU-211, HU-308 and HU-580. Thus:

(i) HU-210 is a synthetic analogue of THC, and a *trans* isomer, which behaves as a highly potent, high-efficacy CB_1_ and CB_2_ cannabinoid receptor agonist [8];
(ii) HU-211 is the synthetic *cis* enantiomer of HU-210, and displays much less activity than HU-210 as a cannabinoid receptor agonist [8];
(iii) HU-308 displays much greater affinity for CB_2_ than for CB_1_ cannabinoid receptors, and is a CB_2_-selective agonist [8];
(iv) HU-580 is a synthetic methyl ester of the phyocannabinoid, cannabidiolic acid (CBDA), which displays greater potency and a broader bell-shaped dose-response curve than CBDA (or cannabidiol) as an enhancer of the activation of 5-HT_1A_ serotonergic receptors and as an inhibitor of signs of anxiety and of chemotherapy-induced nausea in rats, and that is much more stable and so much more “druggable” than CBDA [20].

In conclusion, throughout his research career in the cannabinoid field, Raphael Mechoulam has demonstrated time and again an amazing ability both to come up with exciting original and important ideas that have greatly helped to advance knowledge about cannabinoid preclinical and clinical pharmacology, biochemistry and medicinal chemistry, and to follow these ideas through with great effect. This is due not least to his ability (i) to set up highly talented research teams in his laboratory, to think both as a chemist and as a biologist—a powerful combination—(ii) to recognize how his ideas/discoveries might be exploited in the clinic, and then (iii) to set up productive collaborations with clinicians, with preclinical pharmacologists/biologists, and with pharmaceutical companies. In addition, it is noteworthy that his surname is often used to name conference keynote lectures (e.g., “Mechoulam Lecture”), and to name prestigious awards such as the International Cannabinoid Research Society’s “Mechoulam Award”. He has himself, of course, received many outstanding awards (see https://en.wikipedia.org/wiki/Raphael_Mechoulam), and has authored or co-authored many excellent peer-reviewed papers to the extent that he has received “Highly Cited Researcher Awards” (e.g., from Clarivate Analytics) in recognition of him ranking among the top 1% of researchers in the world for most cited documents in a specific field and particular year. I wish Raphi Mechoulam a very happy 90th birthday!
